# Triple Negative Breast Cancer in an Appalachian Region: Exponential Tumor Grade Increase with Age of Diagnosis

**DOI:** 10.13023/jah.0303.08

**Published:** 2021

**Authors:** Gina Sizemore, Toni Marie Rudisill

**Affiliations:** West Virginia University,

**Keywords:** Appalachia, disease biology, breast cancer testing, genetics, tumor classification

## Abstract

**Introduction:**

Triple negative breast cancer is an aggressive breast cancer with decreased five-year survival, increased risk for recurrence, and higher risk for metastases. Unlike other breast cancers, it has no targeted treatment and has heterogeneous genetics which make classification and treatment difficult.

**Purpose:**

The purpose of our research was to compare triple negative breast cancer to non-triple negative breast cancer to identify key epidemiologic factors that might lead to improved basic science directives for biomarkers, treatments, and classification.

**Methods:**

The state cancer registry was used to provide the first West Virginia state-wide population evaluation of triple negative breast cancer.

**Results:**

The research reveals novel results that tumor grade increases exponentially with the age at diagnosis.

**Implications:**

This creates an epidemiologic foundation for future research to define whether the disease, access to care, biology of aging, or some other factor cause this significant finding. In addition, results reveal decreased use of testing that could be increased to improve biomarker identification, targeted treatments, and classification of triple negative breast cancer.

## INTRODUCTION

Worldwide, breast cancer is a leading cause of cancer mortality in women^1^ and in the U.S., invasive breast cancer impacts 12.4% of the population of women.^2^ Breast cancer is a diverse disease with five molecular subtypes. One type is triple negative breast cancer (TNBC), a breast cancer where tumors are negative for estrogen receptor, progesterone receptor, and Human Epidermal Growth Factor Receptor 2 overexpression.^3,4^ Because of its heterogeneity, TNBC is further divided into sub-types whose classification is still controversial with the most recent being the genetic profile classification.^5^ TNBC is an aggressive disease and is associated with a poorer prognosis and 5-year survival rate as there is a higher risk for recurrence and metastasis among affected patients.^6–8^ Previous studies have shown that the prevalence of TNBC is higher in some demographic groups such as women under 40 years of age and among women of black race or Hispanic ethnicity.^6,7,9–14^

The reasons why certain demographic groups are more affected than others are unknown. Previous studies have proposed that obesity, diet, genetics, socioeconomic, and biological factors may explain differences seen among demographic groups in terms of TNBC.^3,6–8,11,15,16^

Because of the severity of TNBC, there has been an increased interest in investigating demographic, diagnostic, and prognostic factors associated with the disease not only nationwide, but also in West Virginia.^8,9,17^ The age-adjusted breast cancer mortality rate among women in West Virginia is the 8th highest among states.^18^ In addition to the high mortality rate, West Virginia’s population is somewhat homogenous compared to other states as over 94% of the population is non-Hispanic white^19^; thus, racial differences may be observed to a lesser extent. However, the population is of lower socioeconomic status and has greater levels of obesity compared to most other states.^20,21^ The state also has a low net migration of residents which could indicate that hereditary factors associated with TNBC, such as BRCA mutations, may remain present in the population and influence the disease’s prevalence.^3,22^

To date, two studies have investigated TNBC in West Virginia.^8,17^ One study found that West Virginia patients had increased representation of advanced tumors at time of diagnosis.^17^ The other study determined that a greater proportion of women with TNBC were under the age of 50 and that their tumors were larger than non-TNBC patients; TNBC patients also were slightly more obese than non-TNBC patients.^8^ However, both studies utilized unique patient populations from a university hospital and/or a regional medical center over 15 years ago. Thus, the purpose of this study was to investigate the demographic and diagnostic differences between those diagnosed with TNBC compared to non-TNBC utilizing more recent data from the state’s cancer registry, which includes all patients diagnosed with breast cancer in the state. The findings of this study could help inform future research in a state where cancer risk and mortality are high.

## METHODS

### Data Source

The primary data source for this analysis was the West Virginia Cancer Registry, which is maintained by the West Virginia Department of Health and Human Resources in Charleston, WV. Since 1993, the registry maintains demographic and clinical data on individuals who are diagnosed and treated for cancer within the state. The registry also includes West Virginia residents who were treated outside the state boundary but retain a West Virginia address.^23^ The registry collects, codes, and maintains these data in accordance with the National Cancer Institute’s Surveillance, Epidemiology and End Results Program (SEER).^24^ West Virginia follows the guidelines established by their Standards Setting Organization and uses multiple resources including but not limited to SEER Guidelines: North American Association of Central Cancer Registries (NAACCR), Acute Oncology Service (AOS), Commission on Cancer (COC), National Program of Cancer Registries (NPCR). WV provides access to these multiple resources as tools to assist in data collection.

### Study Population

The study population included all West Virginia women who were diagnosed with breast cancer (e.g., International Classification of Diseases for Oncology, Third Edition, codes C500–C509) from January 1, 2010, through December 31, 2016. The data years 2010–2016 were selected because TNBC had not been properly characterized as a separate type of cancer prior to 2010, and 2016 was the most recent data year available.

### Human Subject’s Protections

This study was approved by West Virginia University’s Institutional Review Board (protocol #1908679407).

### Variables

The primary dependent variable was whether an individual was diagnosed with TNBC (e.g., dichotomous). TNBC was defined in accordance with SEER as a breast cancer that is negative for estrogen receptors, progesterone receptors, and Human Epidermal Growth Factor Receptor 2 overexpression.^24^ Various independent variables were utilized for this analysis; several variables were based on Collaborative Stage Site-Specific Factors (CSSSF). The variables included patient’s age at diagnosis, race, year of diagnosis, stage of cancer at diagnosis, whether their cancer metastasized (binary; CSSSF-20), whether the patient’s ipsilateral axillary lymph nodes were implicated (binary; CSSSF-19), whether the cancer was entirely in situ (binary; CSSSF-3), tumor grade (CSSSF-7), whether a multigene test was performed on the patient (binary; CSSSF-22), and whether the patient was diagnosed with Paget’s disease of the breast (binary; CSSSF-24). The categorization of these variables is shown in Table 1. Race was dichotomized into white or other due the demographics of the state (e.g., it is primarily non-Hispanic white).^19^ For tumor grade, some patients were given a Bloom-Richardson score. Those with scores 3–5 were considered low grade. Those with scores 6–7 or 8–9 were categorized as moderate and high grade, respectively.

## ANALYSES

Because the objective of this study was to compare the characteristics of women in West Virginia who were diagnosed with TNBC to other types of breast cancers, several analyses were conducted. The demographic and diagnostic characteristics between those diagnosed with and without TNBC were compared via frequencies and percentages. Characteristics were also compared statistically using Cochran Armitage trend tests with MODRIDIT scoring (for ordinal variables) and Chi Square tests and/or Fisher’s Exact tests (for binary, categorical variables), which was dependent on cell count. In order to determine which variables were associated with TNBC in patients, both binary and multivariable logistic regression analyses were conducted; these types of models were chosen because the outcome was dichotomous.^25^ Univariate (i.e., binary) models were run between each independent variable and the outcome (N=8). All multivariable models were adjusted for age group, race, and year of diagnosis (N=8). (However, it should be noted that the multivariable model for age group was only adjusted for year and race and the multivariable model for race was only adjusted for year and age group). These models were adjusted for these variables because there are known differences with TNBC diagnoses among different age groups and races in other clinical populations.^9^ Year was also adjusted for because of the increasing awareness of TNBC in the literature overtime which could potentially influence diagnoses.^26^ A third set of multivariable models (N=8) were run to investigate potential effect measure modification. These models contained the same variables in the first multivariable model but also included two interaction terms; there was one interaction term between the independent variable of interest and age group and another interaction term between the independent variable of interest and race.^27^ All data management and statistical analyses were conducted in SAS/STAT software version 9.4 (Cary NC) with two-sided significance level α=0.05.

## RESULTS

Nearly 13% of the women diagnosed with breast cancer in West Virginia had TNBC (Table 1). While nearly 60% of all breast cancer patients were over 60 years of age, a slightly larger proportion of women ≤40 years of age were diagnosed with TNBC (7%) compared to those with non-TNBC (4%). In regard to race, there was an increased percentage of non-whites (6%) diagnosed with TNBC compared to the non-TNBC group (3%). The stage of cancer at diagnosis confirmed a more aggressive cancer in TNBC with (4.2%) involving direct and lymph nodes compared to non-TNBC (2.7%) and a distant cancer in TNBC (8.1%) compared to distant cancer in non-TNBC (5.7%) at time of diagnosis. Metastasis was higher in TNBC (6.9%) compared to non-TNBC (5.4%). Tumor grade differed in TNBC patients with 73.1% in high grade compared to only 22.6% high grade in non-TNBC. While only 26% of patients with breast cancer received a multigene test, only 6% of TNBC patients received it compared to 29% of non-TNBC patients.

Table 2 shows the association between TNBC and demographic and diagnostic criteria. After adjusting for race and year, the odds of TNBC diagnoses in women ≤40 was 2.2 times greater than the odds of TNBC in women ≥71 years of age. The odds of a TNBC diagnosis in non-whites was 71% higher than the odds of TNBC in whites after adjusting for age and year. Additionally, it appeared that TNBC was associated with higher tumor grades. The odds of TNBC was 16 times greater in high tumor grades than the odds of TNBC in women with low tumor grades at time of diagnosis after adjusting for age, race, and year. Yet, the odds of a TNBC diagnoses was 86% lower among those receiving a multigene test compared to the odds of a TNBC diagnoses among those not receiving a multigene test.

Only two interactions were statistically significant. Age was an effect modifier of the relationship between TNBC diagnoses and tumor grade (Table 3). After adjusting for both year and race, the odds of TNBC dramatically increased with more severe tumor grades over the age groups. A female under the age of 40 diagnosed with TNBC had 5 times greater odds of having a high tumor grade compared to the odds of a woman in the same age group diagnosed with TNBC having a low- grade tumor. However, the odds of TNBC for a female ≥71 years of age to be high grade stage was nearly 22 times greater than the odds of a TNBC diagnoses for a female in the same age group having a low-grade tumor.

Age was also an effect modifier of the relationship between TNBC diagnosis and multigene test conductance (Table 4). It appears that the conductance of the multigene test decreases with age. While there were no differences in women ≤40, after adjusting for year and race, the odds of TNBC diagnoses among those aged 41–50 years is 75% lower if multigene test is conducted compared to the odds of a TNBC diagnoses among women of the same age who do not receive the multigene signature test. However, the odds of TNBC diagnoses among those aged ≥71 years is 88% lower if multigene test is conducted compared to the odds of a TNBC diagnoses among women of the same age who do not receive the multigene signature test.

## DISCUSSION

Several important findings were discovered as a result of this analysis. First, trends in TNBC diagnoses typically seen in other studies were also seen in West Virginia. Second, TNBC diagnosis and tumor grade varied by age, which is a novel finding. Third, while multigene testing was infrequently performed among all breast cancer patients, there was an inverse relationship between age and multigene conductance among TNBC patients specifically. These are important findings especially when considering the previous literature indicating delayed diagnoses of breast cancer among West Virginia patients.^17^ This generates an imperative to improve early diagnosis, treatment planning, and cancer typing.

In relation to the current literature, the findings were consistent with those of previous nationwide studies. The prevalence of TNBC in West Virginia was 13%, which is similar to the nationwide prevalence of 13%.^9^ Similarly, a there was a larger portion of patients under 40 years old within the TNBC population when compared to the non-TNBC population. Also consistent with the literature was the increased percentage of non-whites in the TNBC population despite a 94% white non-Hispanic population prevalence in West Virginia,^9,19^ and confirmation of TNBC’s aggressive form and increased risk for metastasis. However, the majority of TNBC patients were older adults and there was a significant increased risk for high grade tumor in older patients at time of diagnoses with TNBC.

The novel finding concerning TNBC diagnosis and tumor grade variation by age provides opportunity for future research. The numbers are significant and may be due to biological reasons such as decreased physiologic response with aging, access to care, a feature of the TNBC disease, or some other factor. The finding warrants additional investigation, perhaps using national data.

Multigene testing was not performed frequently, especially in TNBC patients, and the test’s frequency decreased with patient age. The cost, difficulty of coverage for testing, and limitations of many tests in healthcare make this result unsurprising. However, the increasing benefit of genetic and epigenetic research as well as improvements to the multigene test itself may indicate opportunity for change. The data of this project indicates there may be opportunity for improved patient outcomes with future research to identify barriers to its clinical use.

New multigene signature testing provides American Joint Committee on Cancer (AJCC) staging and molecular subtyping, both valuable tools for improving our ability to classify cancer types and generate research data for identifying targeted treatment. Research by Lehman et al discovered from retrospective pretreatment biopsies that prediction regarding neoadjuvant response to therapy was not only possible but indicated the probability that there were both chemotherapeutic sensitive and chemotherapeutic insensitive subtypes in TNBC.^28^ In addition, research from multiple sources of basic science have linked cancer from the breast with the upregulation of a gene, protein, or general pathway.^29^ For example, in 2009, there was documentation of upregulation of hexokinase 2 in breast cancer brain metastasis linked with poor prognosis.^30^ Thus, there is indication that multigene testing could assist evaluation of tumor to aid typing and prognosis for future breast cancer patients. Multigene signature testing alone is not the complete answer to identify targeted treatment, stratify risks, and improve outcomes of clinical applications. However, improved use of this tool in conjunction with other clinical tools and parameters may improve patient stratification for treatment and lead to data for identifying targeted treatments.

### Limitations

While this study highlighted some important differences between TNBC and non-TNBC in West Virginia, it is not without limitation. One of the inherent limitations of this study was that only a limited number of variables were available for analyses. Some diagnostic variables, such as response to neoadjuvant therapy or risk of recurrence, contained a large amount of missing data and could not be analyzed. Also, variables which could impact both the diagnoses and prognoses of patients, such as patient obesity status, insurance coverage, patient preference, comorbidities, or access to care, were unavailable, and could be potential confounding factors.

## CONCLUSION

This research is broad in its evaluation by use of the entire West Virginia breast cancer registry data. It is unique in its analysis to identify present evidence of TNBC disease in West Virginia and its discussion of testing practice to raise pertinent questions. It is translational in its combination of basic science and clinical application perspective.

Triple negative breast cancer in West Virginia is similar to the nation in its demographic evidence and its aggressive nature. This new finding of TNBC diagnosis and tumor grade variation by age urges future research. While the discussion of multigene signature testing reflects opportunity to evaluate policy and practice for its use. Increased efforts are being made to extend multigene testing to whole genome evaluation of tumors to bolster the information gleaned from tumors.^31^ Therefore, increased use of the multigene signature tool may improve our ability to develop biomarkers for early identification of disease, targeted treatment, and response to therapy. Evaluation regarding this finding of exponential increase of high-grade tumor findings with increased age at diagnosis and evaluation of constraints impacting the use of the multigene signature test could be beneficial epidemiologic ventures for future research. This research supports the need for increased focus on tumor evaluation and early diagnosis to improve outcomes for patients.

## Figures and Tables

**Table 1.** Characteristics of West Virginia women diagnosed with triple negative breast cancer compared to those diagnosed with other breast cancer types, 2010–2016^a^

	TNBC (N=1166)		Non-TNBC (N=7934)		Total (N=9100)		P-value
Characteristic	N	%	N	%	N	%	
**Age group**							**<0.0001**
≤40	81	7.0	318	4.0	399	4.4	
41–50	205	17.6	1055	13.3	1260	13.9	
51–60	290	24.9	1832	23.1	2122	23.3	
61–70	317	27.2	2326	29.3	2643	29.0	
≥71	273	23.4	2403	30.3	2676	29.4	
**Race**							**<0.0001**
White	1098	94.2	7665	96.7	8763	96.3	
Other	68	5.8	264	3.3	332	3.7	
**Year of diagnosis**							**0.0255**
2010	167	14.3	979	12.3	1146	12.6	
2011	171	14.7	1052	13.3	1223	13.4	
2012	161	13.8	1138	14.3	1299	14.3	
2013	172	14.8	1172	14.8	1344	14.8	
2014	151	13.0	1166	14.7	1317	14.5	
2015	185	15.9	1197	15.1	1382	15.2	
2016	159	13.6	1230	15.5	1389	15.3	
**Stage at diagnosis**							**0.0056**
Local	734	63.4	5258	66.7	5992	66.2	
Regional direct	26	2.3	151	1.9	177	2.0	
Regional lymph	255	22.0	1817	23.0	2072	22.9	
Direct and lymph	49	4.2	214	2.7	263	2.9	
Distant	94	8.1	448	5.7	542	6.0	
Unknown	8		46		54		
**Metastasis**							**0.0469**
Yes	73	6.9	389	5.4	7867	94.5	
No	993	93.1	6874	94.6	462	5.6	
Missing	100		671		771		
**Node involvement**							**0.9006**
Yes	283	29.8	1985	29.6	2268	29.6	
No	668	70.2	4730	70.4	5398	70.4	
Missing	215		1219		1434		
**In situ status**							
Yes	3	0.3	15	0.2	18	0.2	0.4879
No	1093	99.7	7599	99.8	8692	99.8	
Missing	70		320		390		
**Tumor grade**							**<0.0001**
Low	60	5.6	2164	29.2	2224	26.3	
Moderate	226	21.3	3571	48.2	3797	44.8	
High	777	73.1	1670	22.6	2447	28.9	
Missing	103		529		632		
**Multigene signature test performed**							**<0.0001**
Yes	49	5.8	1759	28.8	1808	26.0	
No	802	94.2	4346	71.2	5148	74.0	
Missing	315		1829		2144		
**Paget’s Disease**							**0.3676**
Yes	6	0.7	62	1.0	68	1.0	
No	900	99.3	5960	99.0	6860	99.0	
Missing	260		1912		2172		

a: The multigene signature test is an assay of a panel of genes that is conducted on a patient’s biopsy. Over 90% of all patients, regardless of breast cancer type, used the Oncotype DX panel, while others used other panels.

**Table 2.** The association between triple negative breast cancer (TNBC) and demographic and diagnostic criteria (N=9100)^a^

	Total N	% of population with TNBC	Univariate Models		Multivariable Models	
Characteristic			OR	95% CI	OR	95% CI
**Age group**						
≤40	399	20.3	2.24	1.70, 2.95*	2.19	1.67, 2.88*
41–50	1260	16.3	1.71	1.41, 2.08*	1.68	1.38, 2.05*
51–60	2122	13.7	1.39	1.17,1.66*	1.38	1.15, 1.64*
61–70	2643	12.0	1.20	1.01, 1.42*	1.20	1.01, 1.42*
≥71	2676	10.2	1.00	Referent	1.00	Referent
**Race**						
White	8763	12.5	1.00	Referent	1.00	Referent
Other	332	20.5	1.80	1.38, 2.37*	1.71	1.30, 2.26*
**Node Involvement**						
Yes	2268	12.5	1.01	0.87, 1.17	0.95	0.81, 1.10
No	5398	12.4	1.00	Referent	1.00	Referent
**In situ**						
Yes	18	16.7	1.39	0.40, 4.81	1.58	0.46, 5.49
No	8692	12.6	1.00	Referent	1.00	Referent
**Tumor grade**						
Low	2224	2.7	1.00	Referent	1.00	Referent
Moderate	3797	6.0	2.28	1.71, 3.05*	2.27	1.70, 3.03*
High	2447	31.8	16.78	12.81, 21.98*	16.24	12.38, 21.30*
**Metastasis**						
Yes	462	15.8	1.30	1.00, 1.68*	1.29	0.99, 1.67
No	7867	12.6	1.00	Referent	1.00	Referent
**Multigene test**						
Yes	1808	2.7	0.15	0.11, 0.20*	0.14	0.11, 0.19*
No	5148	15.6	1.00	Referent	1.00	Referent
**Paget’s disease**						
Yes	68	8.8	0.64	0.28, 1.49	0.66	0.28, 1.53
No	6860	13.1	1.00	Referent	1.00	Referent

* Indicates statistical significance at p≤0.05

Abbreviations: CI=confidence interval; OR=odds ratio; TNBC=triple negative breast cancer Univariate models (N=8) were conducted to assess the relationship between TNBC status (yes/no) and the characteristic. All multivariable models (N=8), except age group and race, were adjusted for age group, race, and year of diagnosis. The multivariable model for age group was adjusted for only year and race. The multivariable model for race was only adjusted for year and age group.

**Table 3.** Age as an effect modifier of the relationship between triple negative breast cancer status and tumor grade^a^

	Model	
Tumor grade by age group	OR	95% CI
≤40		
Low	1.00	Referent
Moderate	1.16	0.36, 3.73
High	5.41	1.85, 15.80*
41–50		
Low	1.00	Referent
Moderate	1.12	0.55, 2.27
High	10.48	5.67, 19.39*
51–60		
Low	1.00	Referent
Moderate	1.89	1.03, 3.46*
High	14.61	8.37, 25.52*
61–70		
Low	1.00	Referent
Moderate	2.54	1.46, 4.42*
High	20.17	11.96, 34.02*
≥71		
Low	1.00	Referent
Moderate	3.64	2.04, 6.50*
High	21.73	12.42, 38.03*

* Indicates statistical significance at p≤0.05

Abbreviations: CI=confidence interval; OR=odds ratio

a: This model was adjusted for year and race. The outcome was whether the patient was diagnosed for triple negative breast cancer vs. regular breast cancer. The primary independent variable was tumor grade at diagnosis, which was stratified by age group

**Table 4.** Age as an effect modifier of the relationship between triple negative breast cancer status and multigene test conductance^a^

	Model	
Multigene test conductance by age group	OR	95% CI
≤40		
Yes	0.52	0.24, 1.13
No	1.00	Referent
41–50		
Yes	0.25	0.15, 0.43*
No	1.00	Referent
51–60		
Yes	0.05	0.02, 0.11*
No	1.00	Referent
61–70		
Yes	0.11	0.07, 0.19*
No	1.00	Referent
≥71		
Yes	0.12	0.04, 0.32*
No	1.00	Referent

* Indicates statistical significance at p≤0.05

Abbreviations: CI=confidence interval; OR=odds ratio

a: This model was adjusted for year and race. The outcome was whether the patient was diagnosed for triple negative breast cancer vs. regular breast cancer. The primary independent variable was whether the patient received multigene therapy, which was stratified by age group

## SUMMARY BOX

### What is already known about this topic?

Triple negative breast cancer is known to be an aggressive breast cancer subtype with a worse prognosis when compared to other breast cancer types.

### What is added by this report?

This report is the first evaluation and analysis of TNBC using data representative of all of West Virginia. This report identifies a new finding of TNBC diagnosis and tumor grade variation by age in this Appalachian population. This report also identifies variability in the use of multigene testing in the TNBC population.

### What are the implications for future research?

Implications for future research are: (1) to identify if this new tumor grade finding is unique to the West Virginia Appalachian population by comparison analysis with nationwide data, and (2) to evaluate policy and practice of multigene signature testing and reporting in West Virginia.
